# Respiratory disease patterns in rural Western Uganda, 2019–2022

**DOI:** 10.3389/fped.2024.1336009

**Published:** 2024-04-08

**Authors:** Taylor E. Weary, Patrick Tusiime, Shamilah Tuhaise, Juan Francisco Mandujano Reyes, Elizabeth Ross, James E. Gern, Tony L. Goldberg

**Affiliations:** ^1^ Department of Pathobiological Sciences, University of Wisconsin School of Veterinary Medicine, Madison, WI, United States; ^2^ The Kasiisi Project, Fort Portal, Uganda; ^3^ Department of Statistics, University of Wisconsin-Madison, Madison, WI, United States; ^4^ Department of Pediatrics, University of Wisconsin Hospital and Clinics, Madison, WI, United States

**Keywords:** respiratory symptoms, pediatrics, cohort study, Africa, COVID-19

## Abstract

**Introduction:**

Respiratory disease is a major cause of morbidity and mortality in the developing world, but prospective studies of temporal patterns and risk factors are rare.

**Methods:**

We studied people in rural Western Uganda, where respiratory disease is pervasive. We followed 30 adults (ages 22–51 years; 534 observations) and 234 children (ages 3–11 years; 1,513 observations) between May 2019 and July 2022 and collected monthly data on their respiratory symptoms, for a total of 2,047 case records. We examined associations between demographic and temporal factors and respiratory symptoms severity.

**Results:**

The timing of our study (before, during, and after the emergence of COVID-19) allowed us to document the effects of public health measures instituted in the region. Incidence rates of respiratory symptoms before COVID-19 lockdown were 568.4 cases per 1,000 person-months in children and 254.2 cases per 1,000 person-months in adults. These rates were 2.6 times higher than the 2019 global average for children but comparable for adults. Younger children (ages 3–6 years) had the highest frequencies and severities of respiratory symptoms. Study participants were most likely to experience symptoms in February, which is a seasonal pattern not previously documented. Incidence and severity of symptoms in children decreased markedly during COVID-19 lockdown, illustrating the broad effects of public health measures on the incidence of respiratory disease.

**Discussion:**

Our results demonstrate that patterns of respiratory disease in settings such as Western Uganda resemble patterns in developed economies in some ways (age-related factors) but not in others (increased incidence in children and seasonal pattern). Factors such as indoor air quality, health care access, timing of school trimesters, and seasonal effects (rainy/dry seasons) likely contribute to the differences observed.

## 1 Introduction

Respiratory disease is a major cause of morbidity and mortality worldwide, especially among young children (1, 2). In low- and middle-income countries, lower respiratory infections rank as the largest cause of mortality in children under 10 years (1), but upper respiratory infections (URIs) (2) and chronic noncommunicable respiratory diseases, such as asthma (3), are significant and growing concerns. Here we use the term “respiratory disease” to encompass all respiratory illness etiologies. In temperate regions, URIs exhibit seasonal patterns that have been attributed to increased viability of pathogens under certain meteorological conditions and increased transmission due to social factors such as school sessions and indoor crowding (4). Few comparable data on respiratory disease epidemiologic patterns exist, however, from resource-limited tropical settings. In Sub-Saharan Africa, for example, URI data derive mainly from influenza surveillance studies of people admitted to hospitals for treatment (5–7). According to these studies, seasonal patterns of respiratory infections are not as pronounced in the tropics as in temperate regions, with outbreaks occurring throughout the year (8, 9). Noninfectious respiratory diseases, such as asthma, may be frequently underdiagnosed in Sub-Saharan Africa (10), but current epidemiological data show that asthma prevalence among children in this region is similar to or higher than global averages (11).

We conducted a prospective cohort study of young children and adults in rural settings in Kabarole District, Uganda. The principal rationale for this study was that there have been few respiratory disease cohort studies in rural Sub-Saharan Africa, which hampers geographic comparisons. Our research objective therefore was to characterize epidemiological patterns of respiratory symptoms in this population and examine potential demographic and environmental risk factors. We obtained records of participants' respiratory disease symptoms at their schools, work, or homes from May 2019 to July 2022. Fortuitously, the study occurred before, during, and after the emergence COVID-19 when public health measures (e.g., closure of schools and businesses, prohibition of travel internationally or between districts in-country, mandatory masking, etc.) were instituted. We were therefore able to capture the changing dynamics of respiratory symptoms during a time of intense public health intervention aimed specifically at reducing respiratory pathogen transmission. We predicted these interventions would result in decreased respiratory illnesses, as has occurred elsewhere in Sub-Saharan Africa (12) and around the world (13). Furthermore, our prospective cohort study design allowed us to examine trends without relying on data from official reporting, which decreased during lockdown restrictions, for example, in the case of tuberculosis (14). Our data illustrate important similarities and differences between patterns of respiratory disease in this developing rural economy and both global averages and averages from high-income countries, particular with respect to overall incidence of disease, age-associated effects, and seasonality.

## 2 Materials and methods

### 2.1 Study sites, subjects, and sample collection

We established a prospective cohort study of schoolchildren (Children from schools: Cs; ages 3–6, *n* = 203) attending three primary schools between 3 and 13 km apart from each other, here abbreviated as KKO, KNS, and RWT. These schools are near a national park that is a focus of international research and a major local employer (15, 16). For comparison, we enrolled 30 adults employed as research assistants at the national park (Ad; ages 22–51) and 31 children of these individuals (Children of adults: Ca; ages 3–11) who attended the same primary schools listed above. These individuals were valuable additions to the study because they lived in the same area as the Cs cohort but likely experienced different socioeconomic conditions, and because they enabled comparison of children and adults from the same households. Due to data de-identification required for institutional ethics approval, the ages of individuals within each group were masked. To estimate sample sizes for regression analyses (see below), we assumed a logistic model with 10 independent variables, and we simulated sample sizes using odds ratios (OR) as effect sizes and performed Wald tests on the simulated data to assess power. We chose a minimum effect size of 3.5 based on recommendations of Chen et al. (17) that OR of 3.47 are equivalent to Cohen's d of 0.5 (medium effect) when disease frequencies are approximately 1%. Thus, a sample size of 114 (or 38 per cohort) would be adequate to detect a medium effect. However, our actual sample sizes were larger due to our repeated measures design and better-than-expected participation rates, and disease frequency was higher than 1% (see below), which gave us adequate statistical power.

Data on respiratory symptoms [cough, runny nose (rhinitis), sore throat, sneezing, wheezing, dyspnea, and fever] and severity of cough and runny nose were collected monthly from May 2019 to August 2021 by Ugandan healthcare professionals (registered nurses). Nurses sought informed consent then surveyed consenting participants about respiratory symptoms they had experienced in the previous two weeks in Rutooro, the local language, of which the nurses were native speakers. The survey instrument was adapted from those used in two birth cohort studies in the US (18, 19) (Supplementary Figure S1), and we piloted it on other native Rutooro speakers before deploying it. Points were scored as follows: 1 point each was assigned for fever, mild (dry) cough, mild runny nose, sore throat, and illness duration of ≥4 days; 2 points were assigned for moderate cough (productive cough but subject sleeps through the night) or moderate-to-severe runny nose (nose needs to be wiped once an hour or more); 3 points were assigned for severe cough (productive cough that wakes subject at night or vomit with cough); and 5 points were assigned for wheezing or dyspnea. For each participant at each time point, we calculated a “symptoms score” as the sum of all points, which ranged from a possible low of zero to a possible high of 19. In calculating incidence rates, we assumed independence between URI cases reported in different months due to the short symptoms duration (7–10 days) of the most common pathogens (20).

Data from the Global Burden of Diseases, Injuries, and Risk Factors Study (GBD) 2019 were used for comparison to data collected in this study (1). The GBD 2019 estimates incidence, prevalence, mortality, and disability-adjusted life-years (DALYs) from 369 diseases and injuries, for both sexes, from 204 countries and territories, including Uganda. Data from GBD 2019 are publicly available online and can be extracted using the Global Health Data Exchange query tool (https://vizhub.healthdata.org/gbd-results/). Data sources and inclusion criteria for URIs are described elsewhere (1). The current study is not associated with the GBD Institute for Health Metrics and Evaluation Commission.

**Figure 1:**
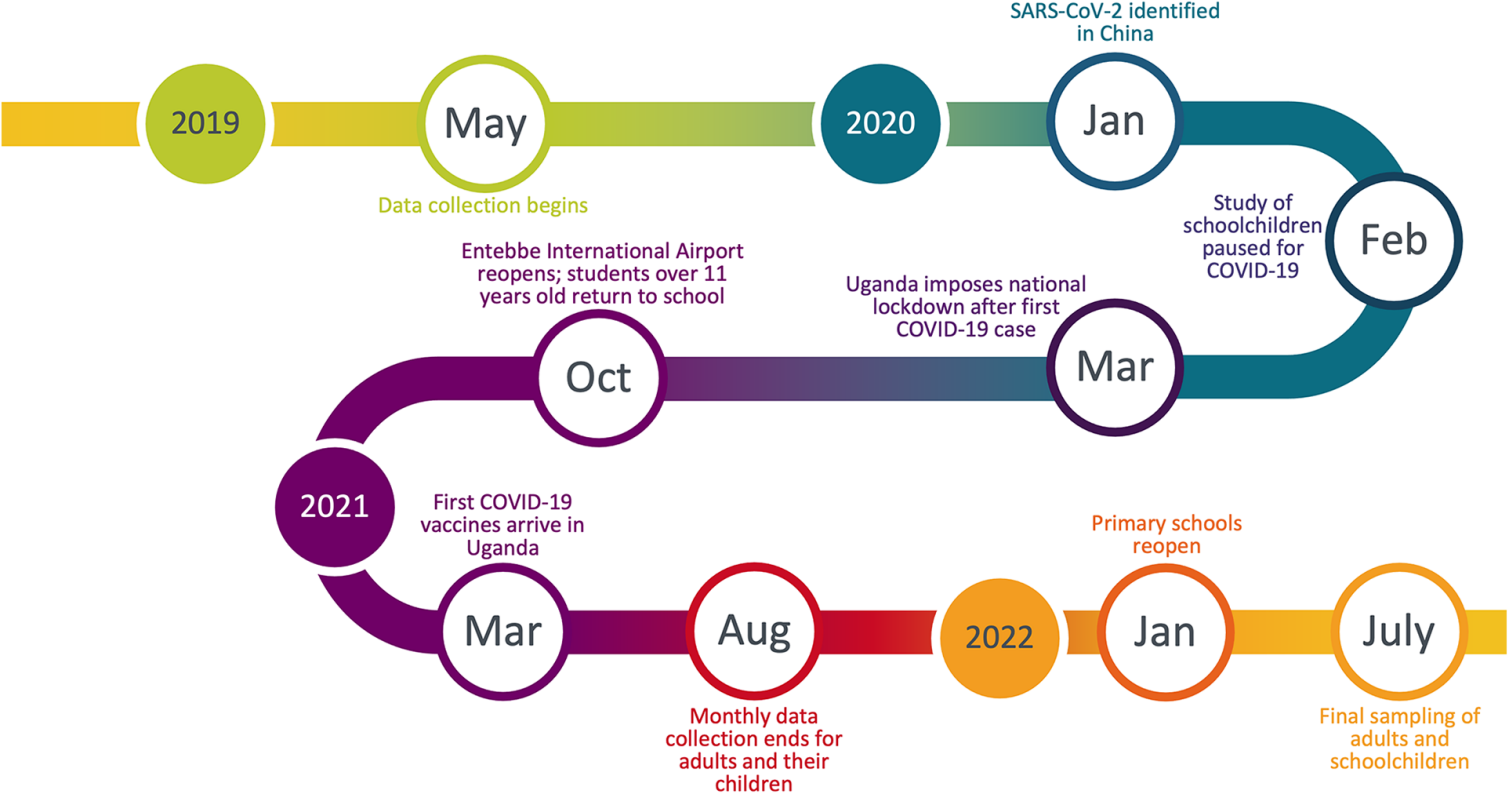
Timeline of prospective cohort study and COVID-19 in Uganda, 2019–2022.

After Uganda's national lockdown was instated on March 20, 2020, schools were suspended, such that we were no longer able to follow the three cohorts of schoolchildren (Cs) until after schools reopened in January 2022 (Figure 1). We therefore obtained permission from the Uganda National Council for Science and Technology, the Makerere University research ethics committee, and study participants themselves to continue sampling adults (Ad) and their children (Ca) at their homes throughout this period. We collected data from these individuals monthly until August 2021, after which sampling was paused until after schools reopened. Data collection during this period followed strict biosafety protocols from the Uganda National Council for Science and Technology to protect both study participants and researchers from COVID-19. In July 2022, we collected a final round of data on children from the three schools (Cs) and adult participants (Ad) to capture changes in respiratory symptoms frequency that may have occurred following the reopening of schools and the loosening of COVID-19 restrictions on travel and businesses. Children of adult participants (Ca) were not accessible at this time because many had changed schools and the rest declined participation because of the long hiatus.

### 2.2 Inferential statistics

Incidence rates and relative risk (RR) were calculated using presence (symptoms scores > 0) or absence (symptoms scores = 0) of symptoms. To examine associations between respiratory symptoms and putative risk factors, we used linear mixed models (LMMs) and generalized linear mixed models (GLMMs) using the lmerTest and lme4 packages, respectively, in R (21, 22). The response variable for the zero-inflated Poisson GLMM was symptoms score (severity), and the response variables for the LMMs and binomial GLMMs were symptoms score (severity) and presence/absence of symptoms, respectively. Parametric model assumptions were assessed with normal probability plots for verification of normality and with Levene's test for verification of homogeneity of variances. Model diagnostics were performed using the DHARMa (23) and lmerTest packages in R. We included subject ID as a random effect to control for multiple sampling of individuals. Significance of full models and of random effects was evaluated using log-likelihood ratio tests. The best models were selected using Akaike's information criteria (AIC) (24). All statistical tests were performed at *α* = 0.05 (two-sided).

To account for missing Cs data after schools shut down due to COVID-19 in March 2020 in analysis of seasonality and other non-COVID-related trends, we employed the Multiple Imputation of Incomplete Multivariate Data method based on the Expectation-Maximization (EM) algorithm, with bootstrapping, in the Amelia II package in R (25), using m = 5 imputed datasets. This approach was used to impute only Cs symptoms scores during lockdown (March 2020–August 2021), comprising 8.6% of the total symptoms scores, by using Ad sample collection dates during lockdown and Cs individual subject IDs.

Average monthly weather data for 1991–2021 available via the European Centre for Medium-Range Weather Forecasts (ECMWF) for Fort Portal, Uganda, included rainfall (mm), rainy days per month, hours of sunlight per day, temperature (°C, including maximum, minimum, and average values), and humidity (%) (26). To account for multicollinearity among these predictors, we employed principal component analysis, a machine learning tool for variable reduction while still maximally capturing data variance (27) using the vegan package in R (28). Humidity and maximum monthly temperature loaded most strongly onto PC1 (Supplementary Table S1), and PC1 and PC2 were ultimately included in our GLMM and LMM with 98.8% total weather variable proportion explained.

## 3 Results

We collected 2,047 case records (symptoms score cards) from 30 adults (534 cards) and 234 children (1,513 cards) between May 2019 and July 2022. Participant demographic information, data collection information, and incidence rates of respiratory symptoms are given in Table 1. Detailed symptoms data are available in Supplementary Table S1.

**Table 1:** Demographic information, data collection information, and respiratory symptoms incidence rates for the three cohorts.

Cohort	Ages (years)	Collection Dates	Participants (*n*)	Symptoms Scores (*n*)	Incidence: Before COVID-19 Lockdown^a^	Incidence: COVID-19 Lockdown^c^	Incidence: After COVID-19 Lockdown^d^
Adults (Ad)	22–51	May 2019–August 2021; July 2022	30	534	254.2	160.8	200.0
Children of adults (Ca)	3–11	June 2019–August 2021	31	477	608.9^b^	256.0	n/a
Schoolchildren (Cs)	3–6	June 2019–February 2020; July 2022	Total: 203 KKO: 62 KNS: 82 RWT: 59	1,036	667.1^b^	n/a	814.0

^a^ Cases per 1,000 person-months (May 2019–March 2020).

^b^ Incidence in all children prior to COVID-19 lockdown: 568.4 cases per 1,000 person-months.

^c^ Cases per 1,000 person-months (April 2020–August 2021).

^d^ Cases per 1,000 person-months (July 2022).

### 3.1 Incidence rates of respiratory illnesses

Prior to COVID-19 in Uganda (May 2019–March 2020), incidence rates of respiratory symptoms (any symptoms scores > 0) were 2.2 times higher in children than in adults (568.4 vs. 254.2 cases per 1,000 person-months, relative risk [RR] 95% CI [1.8, 2.8]) (Table 1). Respiratory disease incidence rates in adults were comparable to previously reported rates for similarly aged adults (20–54 years) globally (254.2 vs. 177.9 cases per 1,000 person-months, RR = 1.4, *X*^2 ^= 2.2, *p* = 0.1416), in Uganda (254.2 vs. 216.9 cases per 1,000 person-months, RR = 1.2, *X*^2 ^= 0.2, *p* = 0.6660), and in high-income countries (254.2 vs. 216.9 cases per 1,000 person-months, RR = 1.2, *X*^2 ^= 0.2, *p* = 0.6660) in 2019 (1, 2). However, rates for children in this study were higher than previously reported rates for similarly aged children (1–9 years) globally (568.4 vs. 220.2 cases per 1,000 person-months, RR = 2.6, *X*^2 ^= 162.2, *p* < 0.0001), in Uganda (568.4 vs. 262.9 cases per 1,000 person-months, RR = 2.2, *X*^2 ^= 110.6, *p* < 0.0001), and in high-income countries (568.4 vs. 345.2 cases per 1,000 person-months, RR = 1.6, *X*^2 ^= 51.1, *p* < 0.0001) (1, 2). Thus, patterns of childhood respiratory disease in Western Uganda differ from those observed elsewhere.

### 3.2 Age and symptom patterns

**Figure 2:**
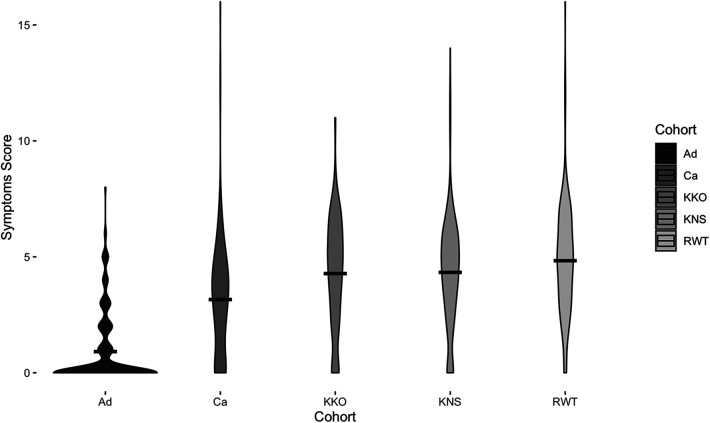
Symptoms scores for each cohort. Width of the violin plot indicates data density at a particular symptoms score. Black bars represent means. Displayed data are from before COVID-19 (May 2019–March 2020) only.

**Figure 3:**
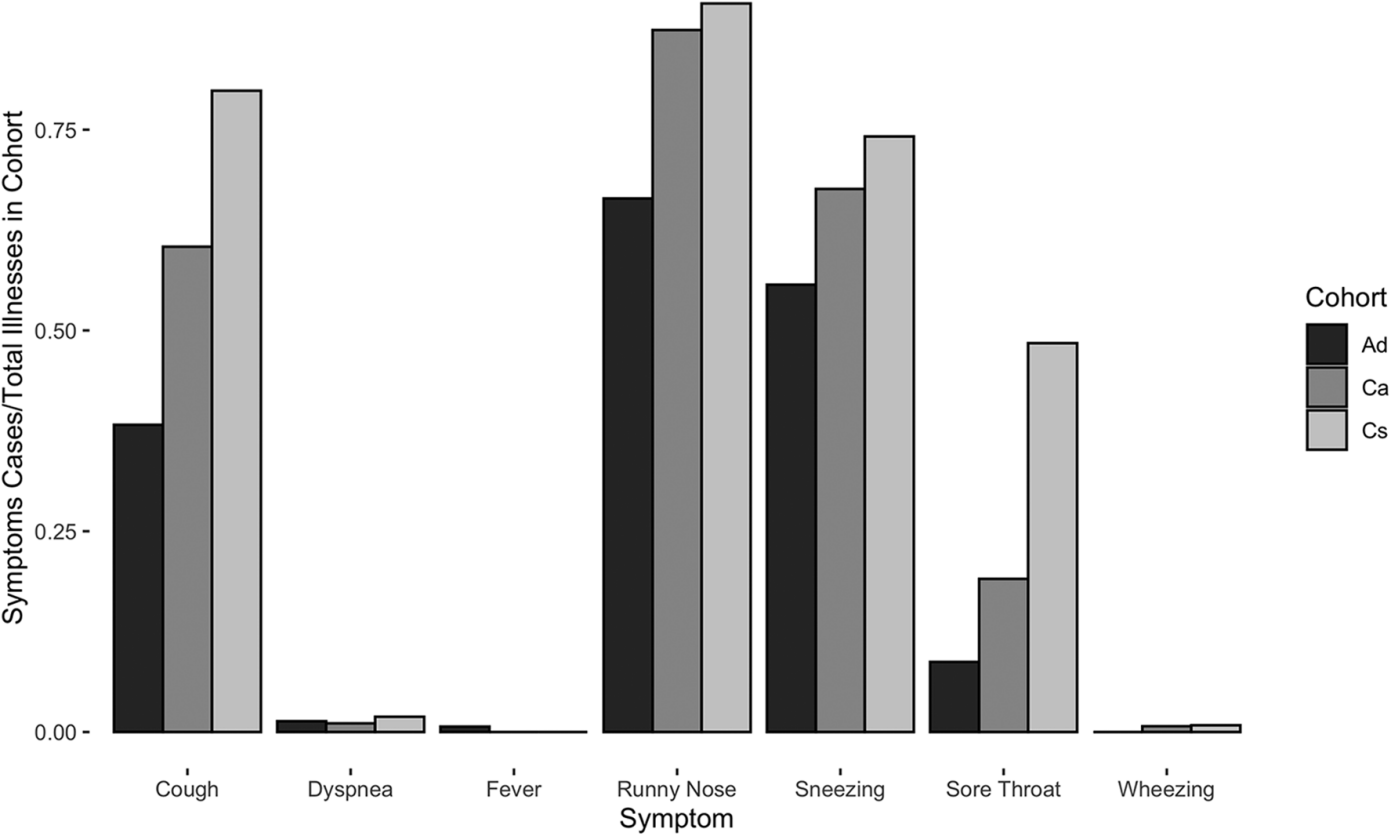
Cases of respiratory symptoms normalized by total illnesses per cohort for Ad, Ca, and Cs cohorts between May 2019 and July 2022.

Children reported higher mean symptoms scores than adults (Welch's *t*-test, t_2716 _= −56.5, *p* < 0.0001) (Figure 2). Children of adult participants (Ca) and schoolchildren (Cs) reported comparable incidence rates before COVID-19 lockdown (RR [95% CI] = 1.0 [0.9, 1.2]). However, Ca had significantly lower mean scores than Cs (Welch's *t*-test, t_2557 _= −39.0, *p* < 0.0001) (Figure 2). Children reported experiencing any respiratory symptoms on 80.3% of score cards, whereas adults reported respiratory symptoms on 26.6% of score cards. Cs had the most symptoms per illness, followed by Ca and Ad (Figure 3). The most frequent respiratory symptom reported in each group was runny nose (Ad: 73.1, Ca: 225.8, Cs: 466.1 cases per 1,000 person-months) while the least frequent was wheezing unrelated to exercise, which only occurred in 10 children (Ad: 0.0, Ca: 2.7, Cs: 4.9 cases per 1,000 person-months). Only one person reported having a fever (an adult on a single occasion). Incidence rates of all symptoms were higher in children than in adults, except for fever (RR [95% CI]: cough = 5.7 [4.5, 7.3], sore throat = 13.8 [8.1, 23.8], sneezing = 24.2 [14.1, 41.5], runny nose = 3.9 [3.3, 4.7], fever = 0.4 [0.0, 5.6], wheezing = 3.5 [0.5, 27.5], and dyspnea = 3.7 [0.9, 15.8]) (Figure 3). Incidence rates were also higher in Cs than in Ca for all symptoms (RR [95% CI] of Cs vs. Ca: cough = 2.1 [1.8, 2.4], sore throat = 4.0 [3.1, 5.2], sneezing = 1.7 [1.5, 1.9], runny nose = 1.6 [1.5, 1.8], wheezing = 1.8 [0.4, 8.7], and dyspnea = 2.8 [0.82, 9.4]). The most frequent type of cough was moderate and productive (52.5% of all reported coughs), followed by mild and dry (30.6%) and severe and productive (17.0%).

Cs experienced prolonged symptoms (≥4 days) significantly more often than did Ca*,* and both cohorts of children experienced prolonged symptoms significantly more often than did adults (Ad: 15.4%, Ca: 28.3%, Cs: 45.8% of all score cards; Welch's ANOVA with Games-Howell post-hoc test, all *p* < 0.0001).

### 3.3 Other factors related to symptoms scores

Children attending RWT Primary School reported significantly higher mean symptoms scores than children at the other two schools (ANOVA with Tukey HSD, *p* = 0.01) (Figure 2). Multivariate regression controlling for time period (before and after COVID-19 lockdown) also showed children attending RWT Primary School were significantly more likely to have reported symptoms, and to have reported more severe symptoms, than children at the other two schools (Table 2). There were no significant differences among the children in three schools in prevalence of respiratory illness bouts lasting ≥4 days (50.0% [KKO], 44.7% [KNS], and 42.8% [RWT]; Welch's ANOVA with Games-Howell post-hoc test, lowest pairwise *p* = 0.0701) (data not shown).

**Table 2:** Results of (a) binomial GLMM for effects of weather/climate variables on schoolchildren's presence/absence of symptoms and (b) LMM for effects of weather/climate variables on schoolchildren's severity of symptoms.

(a) Fixed effects^a^	*β*	SE	Wald *X*^2^ test			*p*
(intercept)	3.0702	0.4134	–			–
KNS^c^	0.1028	0.4323	0.238			0.8120
RWT^c^	1.4665	0.5669	2.587			0.0097**
Weather PC1	−1.0274	0.4316	−2.381			0.0173*
Weather PC2	0.7380	0.4377	1.686			0.0975
Random effect	Variance	SD	Likelihood ratio test (*X*^2^)			*p*
Subject	3.4123	0.3278	16.092			0.0029**
(b) Fixed effects^b^	*β*	SE	95% CI	df	t	*p*
(intercept)	4.2168	0.2060	–	–	–	–
KNS^c^	0.1025	0.2727	[−0.428, 0.602]	188.7	0.376	0.7074
RWT^c^	0.6061	0.3005	[0.036, 1.179]	200.1	2.017	0.0451*
Weather PC1	−0.7550	0.2138	[−1.195, −0.333]	806.6	−3.532	0.0004***
Weather PC2	0.2938	0.2183	[−0.158, 0.706]	842.7	1.346	0.1786

^a^ The marginal and conditional *R*^2^ of the GLMM were 0.074 and 0.505, respectively.

^b^ The marginal and conditional *R*^2^ of the LMM were 0.019 and 0.342, respectively.

^c^ Reference category for cohort: KKO.

*p < 0.01; **p < 0.01; ***p < 0.001.

Individual subject identity was related to symptom scores (GLMM conditional *R*^2^ for presence/absence of symptoms = 0.505; LMM conditional *R*^2^ for symptoms severity = 0.342) (Table 2). However, neither humidity and maximum monthly temperature (which loaded most heavily onto weather PC1 and PC2) nor days since the start of the school term explained a meaningful proportion of the variance in symptoms scores (adjusted *R*^2 ^= 0.0058), and thus were removed from the final model.

### 3.4 Respiratory illnesses during COVID-19

**Figure 4:**
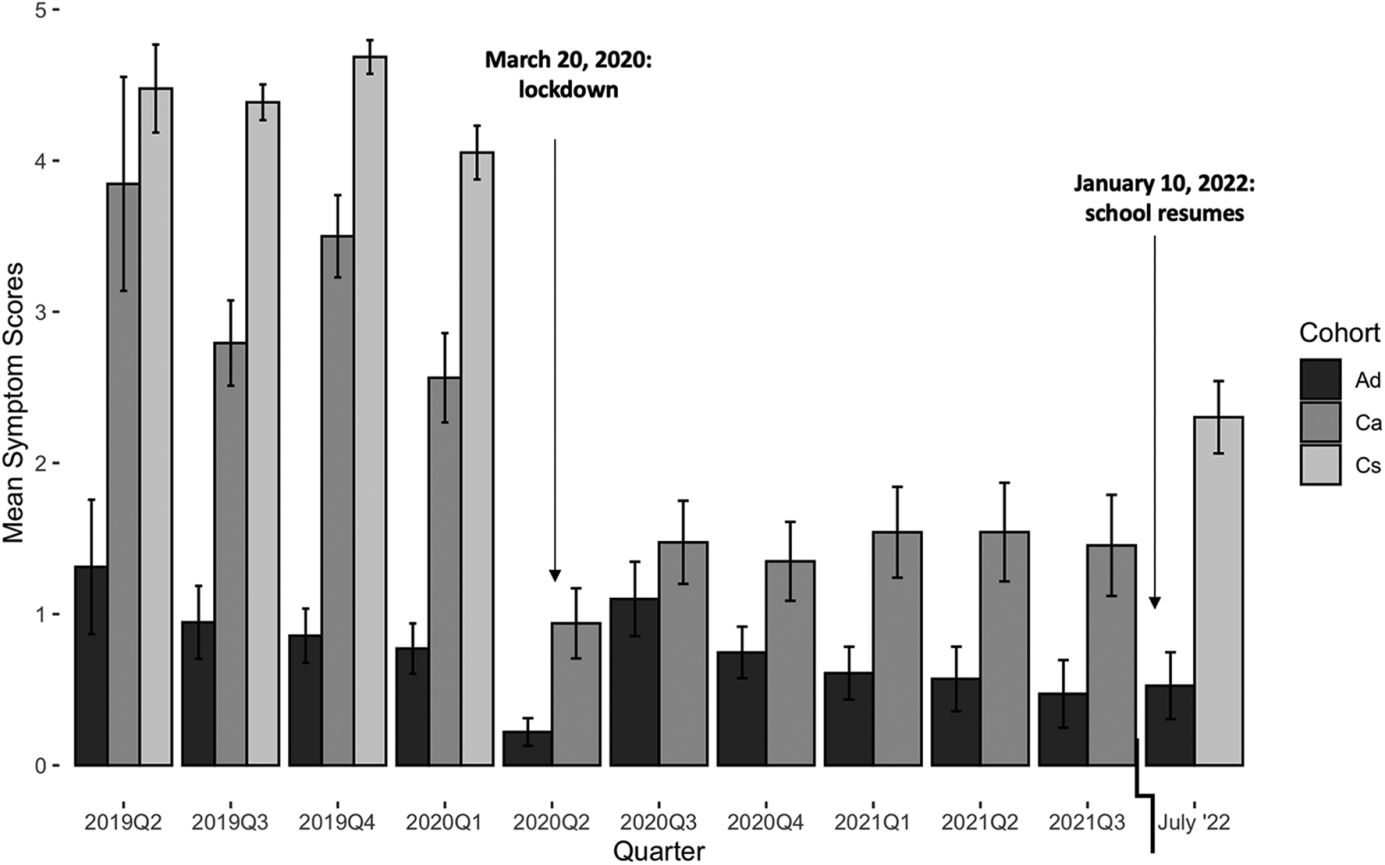
Quarterly mean symptoms scores in Ad, Ca, and Cs cohorts. Bars represent standard errors.

During COVID-19 lockdown, respiratory disease incidence in Ca decreased 2.4-fold [RR 95% CI (2.0, 2.8)], whereas incidence in Ad decreased 1.6-fold [RR 95% CI (1.2, 2.1)]. The mean symptoms score in Ca decreased 2.2-fold (from 3.2 to 1.4; t_332 _= 8.8, *p* < 0.0001), whereas the mean score in Ad decreased 1.7-fold (from 1.0 to 0.6; t_513 _= 2.6, *p* = 0.0095) (Figure 4). In July 2022, after the re-opening of schools and businesses, the mean Ad score (0.5) was indistinguishable from that before or during COVID-19 lockdown (Welch's ANOVA with Games-Howell post-hoc test, lowest pairwise *p* = 0.5070). However, the mean Cs score (2.3) remained lower than before COVID-19 lockdown (4.4) (t_50.7 _= 8.6, *p* < 0.0001). We were unable to sample Ca upon returning in July 2022 due to many of these individuals having changed schools after lockdown.

### 3.5 Seasonality of respiratory illnesses

**Figure 5:**
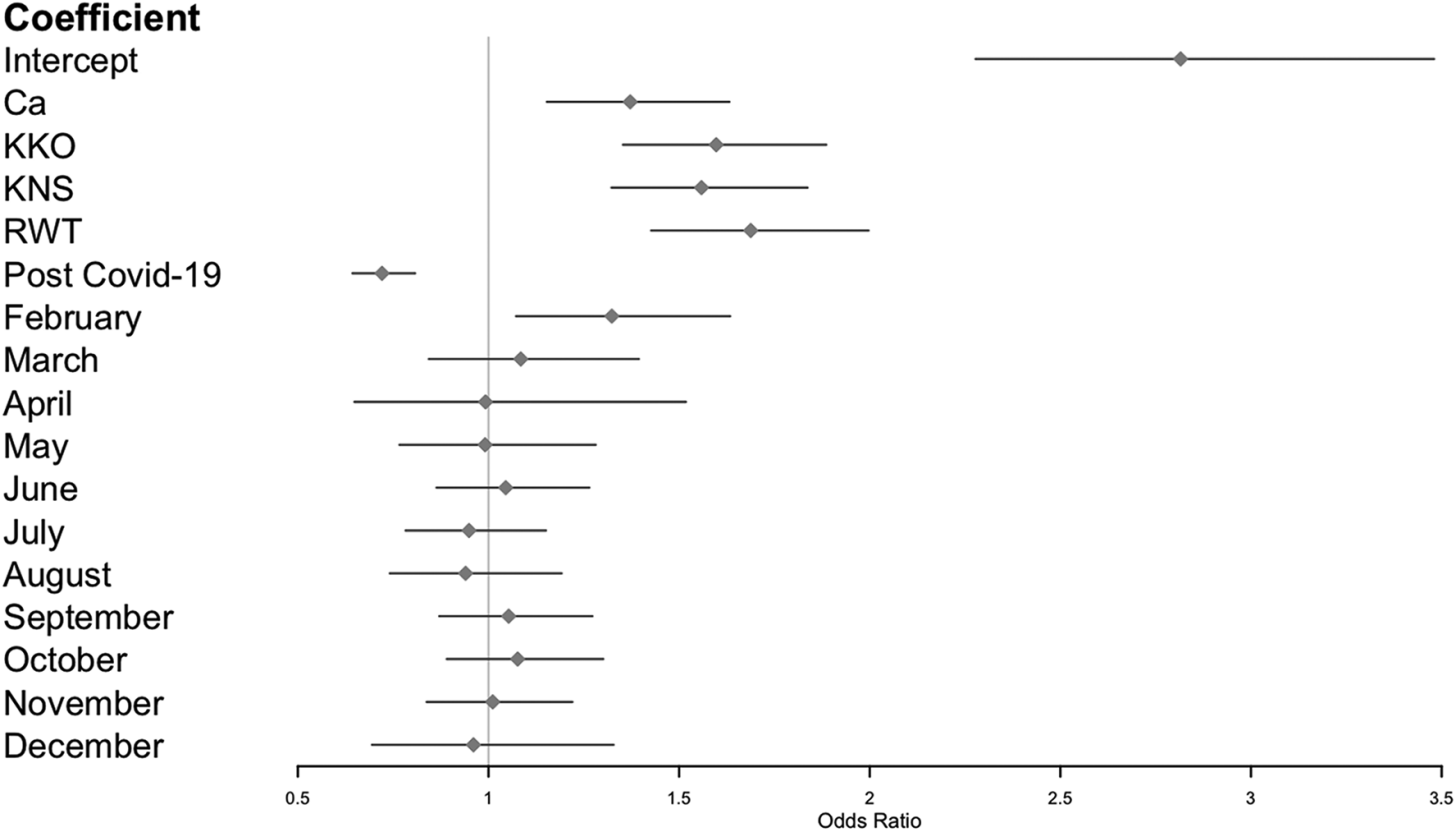
Zero-inflated Poisson GLMM of symptoms scores, including imputed data from schoolchildren during COVID-19 lockdown (see methods). Reference categories: Ad (for cohorts), pre-COVID-19 (for pandemic phase), and January (for months).

Symptoms scores were more likely to be ≥1 in the month of February (Figure 5). No other month of the year displayed a significant trend in either direction.

## 4 Discussion

In this prospective cohort study of people in rural Western Uganda, we characterized epidemiological patterns of respiratory symptoms and examined potential demographic and temporal risk factors for those symptoms. Children experienced respiratory symptoms in 80.3% of monthly samplings and for days or sometimes weeks at a time. By contrast, adults reported respiratory symptoms only 26.6% of the time. Prior to COVID-19 in Uganda, incidence rates of respiratory disease were 568.4 cases per 1,000 person-months in children and 254.2 cases per 1,000 person-months in adults. However, after COVID-19 lockdown measures were implemented, these rates declined to 256.0 and 160.8 cases per 1,000 person-months, respectively. Both before and after COVID-19 lockdown, children experienced more severe respiratory symptoms than did adults. URIs are often caused by pediatric “common cold” pathogens that produce symptomatic disease among children but are asymptomatic in adults, who have been exposed many times throughout their lives (29). Higher severities and longer durations of symptoms in Cs (ages 3–6 years) compared to Ca, who were on average older children (ages 3–11 years), concur with such observations and support the idea that cumulative exposures to URI pathogens lead to enhanced resistance due to adaptive immunity (30). In all age groups, severe respiratory symptoms, such as wheezing, dyspnea, and a productive cough, were uncommon. The low reported incidence of fever among both children and adults (only one case, in an adult) in a malaria-endemic region was unexpected. This may reflect a truly low incidence of fever, or it may have resulted from response bias. For example, the local language, Rutooro, contains no word for “fever” that does not also mean “malaria”. Alternatively, the survey's focus on respiratory illness may have predisposed respondents to report information about fevers experienced in the context of respiratory illnesses. Future studies involving direct measurements of body temperature would be required to clarify this issue. Similarly, the low reported incidence of wheezing and dyspnea could be further investigated through auscultation or respiratory rate measurement.

Children's respiratory symptoms varied with which school they attended. Children attending RWT were more likely to experience any symptoms and to experience more severe symptoms than children attending KKO or KNS. We suspect from observations of conditions at RWT that this difference may be due to overcrowding, dirt floors instead of concrete, and poor access to handwashing supplies. Of the three schools, RWT is also furthest away from the nearest major town (Fort Portal), indicating limited access to health care. These findings suggest that school conditions may contribute to respiratory health of pupils. Unmeasured demographic, behavioral and socioeconomic factors may also have affected our results. For example, while the adults in the study were matched socioeconomically with their children (Ca), they may not be representative of the population of all of Western Uganda due to their employment, which affords more reliable wages than many other local work opportunities, such as subsistence farming, tea plantation work, and casual labor in Fort Portal (16). Future studies controlling for such factors would help disentangle their effects on pediatric respiratory disease in this region.

Respiratory illness rates for children in this study were approximately twice those of previously reported rates for children globally, in Uganda, and in high-income countries in 2019 (2). In contrast, respiratory disease incidence rates in adults were comparable to those reported elsewhere (2). There are several potential explanations for these findings. The GBD 2019 study relied on data from a limited number of URI studies that met their inclusion criteria (1), such that URIs in children may have been underreported in the GBD study. Alternatively, children in our study area may suffer higher rates of respiratory disease than children elsewhere, including in other parts of Uganda. People in our study area are more likely to experience local poverty indicators (e.g., fewer livestock owned, poorer water quality) than people living in other parts of Uganda (31). These socioeconomic conditions are associated with air pollution exposure, comorbidities such as diarrheal disease, and high levels of stress, which could all contribute to increased frequency of respiratory disease. Such effects have been documented for children living in low-income urban areas of the United States (32–34) and Kenya (35), for example.

National lockdown for the COVID-19 pandemic in Uganda included closure of schools, businesses, the international airport, and roads between districts, as well as increased masking and handwashing. Our data indicate that these measures significantly reduced children's respiratory symptoms incidence and symptoms severities. Although the timing of COVID-19 lockdown restrictions varied among countries, similar decreases in respiratory disease during the most stringent parts of lockdown have been observed in other parts of the world, such as Austria (36), South Korea (37), Israel (38), and the United States (13). Mean symptom scores of the schoolchildren in our study were significantly lower in July 2022 after restrictions were lifted than before the pandemic, consistent with continued disruption in non-SARS-CoV-2 respiratory pathogen transmission observed globally (13). Incidence rates and symptoms severities in adults did not change before, during, and after COVID-19 lockdown, likely because these rates are normally low in adults (30, 39). Pre- and post-COVID-19 values for children are not directly comparable in this case because of the absence of the Cs cohort during lockdown and the Ca cohort after lockdown, but these rates still indicate that public health measures targeting COVID-19 reduced respiratory symptoms in children and adults alike, as has been reported elsewhere (13).

Study participants were most likely to experience symptoms in February. To our knowledge, this seasonal pattern has not previously documented. Days since the start of the school term was not a significant predictor of this effect, as might be expected if it were the result of increased contact and transmission. Weather (maximum monthly temperature and humidity) was related to symptoms but explained only a small proportion of variation in the trend. Maximum monthly temperature and humidity loaded in nearly equal and opposite directions in the principal component analysis, suggesting opposing effects of these factors (i.e., increased symptoms scores were associated with higher temperatures and lower humidity) (Supplementary Table S1). In Western Uganda, February is the hottest and driest month of the year (26), which also favors the formation and persistence of airborne particulates (i.e., “dust” from soil and fires) (40), and such conditions may compromise host mucosal defenses (41). Accordingly, dry and dusty conditions each February may contribute to both infectious and non-infectious etiologies of respiratory disease. Intriguingly, multiple participants reported sneezing and coughing “only in the morning” over the course of the study with no discernible seasonal pattern, further suggesting that non-infectious causes, such as smoke from indoor biomass (e.g., charcoal, wood) cookstoves that are lit each morning in the area or perhaps respiratory irritants from housing materials, may contribute to respiratory disease symptoms (42). Similarly, in urban areas of the US, spikes in air pollutants can produce upper and lower respiratory illnesses (34). Respiratory allergies can also provoke non-infectious nasal and chest symptoms in children, but is an unlikely explanation for symptoms in Uganda, where in the city of Entebbe the prevalence of allergic rhinitis was estimated to be <5% (43). However, rural environmental allergens may differ substantially from those in urban areas, warranting further investigation of this potential etiology in our study population.

Interindividual variation influenced respiratory disease rates more than intra-individual variation due to weather or time of year. Individual respiratory disease risk may be influenced by genetic susceptibility, previous pathogen exposure and immunity, social factors, and an individual's environment (44). Unfortunately, de-identification of samples and data precluded analyses of individual factors as modifiers of respiratory disease risk. Future studies in rural Africa of variables such as age, sex, socioeconomic status, household size, exposure to indoor air pollution, and comorbidities would likely prove informative. Other limitations include recall bias in young children [which previous research has shown to be minimal (19, 32)] and loss of the Cs cohort during lockdown and the Ca cohort afterward (see above), but our conservative data imputation strategy (45) helped minimize the latter (46) while still allowing investigation of seasonality and other trends unrelated to COVID-19.

Overall, our results illustrate that patterns of respiratory symptoms in rural areas of developing economies resemble those of developed economies in some ways (e.g., age-related factors) but not in others (e.g., overall incidence and seasonal patterns). Further studies that address the causes of respiratory disease in rural Africa should help explain the trends we have documented. In particular, distinguishing infectious from non-infectious causes of respiratory symptoms, and identifying the etiologies of each, should reveal ecological, epidemiological, and microbiological mechanisms underlying the patterns observed. These mechanisms, in turn, should prove informative for evidence-based public health interventions to reduce respiratory disease incidence in Western Uganda and other similar locations.

## Data Availability

The raw data supporting the conclusions of this article will be made available by the authors, without undue reservation.
