# Supplementary material for: Respiratory disease patterns in rural Western Uganda, 2019–2022
Source: Front Pediatr. 2024 Apr 8;12:1336009. doi: 10.3389/fped.2024.1336009 (PMC11033374; doi:10.3389/fped.2024.1336009)
Supplement: Supplementary file 2 [file Image1.pdf]

Supplementary Figure

S1: Symptoms score data recording instrument.

**Affiliation**
Ad, Ca, Cs [KKO, KNS, RWT]
**In the past two weeks, have you experienced:**
**Cough**
Mild (dry cough) (1 pt)
Moderate (productive but not waking subject at night) (2 pts)
Severe (productive; waking subject at night or vomit with cough**)** (3 pts)
**Sore throat/hoarse** (1 pt)
**Sneezing** (1 pt)
**Runny nose**
Mild (1 pt)
Severe (nose needs to be wiped >1 times per hour) (2 pts)
**Fever** ≥38°C (1 pt)
**Wheezing** not due to exercise (5 pts)
**Dyspnea** (difficulty breathing, chest retractions, etc.) (5 pts)
**Date of onset of illness**: ________________ (*≥4 days = 1 pt)*
**Total point score**: ____________
